# Multi-view dynamic manifold reconstruction with adaptive cross-attention fusion enables noise-robust bearing fault diagnosis

**DOI:** 10.1038/s41598-026-50621-z

**Published:** 2026-04-29

**Authors:** Lei Yang, Liang Zhang, Jun Xu, YuBin Guo

**Affiliations:** 1 Drone Industry College (School of Artificial Intelligence), Chengdu Aeronautic Polytechnic University, Chengdu, 610100 China; 2 CEC Anshi (Chengdu) Technology Co., Ltd, Chengdu, 610100 China

**Keywords:** Bearing fault diagnosis, Phase space reconstruction, Cross-attention, Multi-view fusion, Robustness to intensive noise, Engineering, Mathematics and computing

## Abstract

To address the issues that bearing fault features are easily submerged under strong noise and that manifold topologies are prone to collapse, this paper proposes a Multi-view Dynamic Manifold Reconstruction and Adaptive Cross-Attention Fusion Network (MDM-CA Net). First, based on the theory of phase space reconstruction, dynamic K-nearest neighbor graphs are constructed in both the time domain and the frequency domain. A dual-channel graph convolutional network is employed to mine the intrinsic geometric structure of the signal, while complementary information from multiple views is utilized to filter out spurious connections induced by noise, thereby isolating noise outliers at the representation level. Second, a noise-aware temporal enhancement module centered on a bidirectional gated recurrent unit and integrated with a global attention mechanism is developed to adaptively suppress background noise interference. Finally, a Transformer-style cross-attention strategy is introduced, where temporal features serve as queries to retrieve critical patterns from spatial topologies, enabling deep semantic alignment and nonlinear interaction between heterogeneous spatiotemporal features. Experimental results on three public datasets, CWRU, SEU, and PU, demonstrate that MDM-CA Net achieves optimal comprehensive performance under both same-domain and cross-domain operating conditions. Under the extreme condition with a signal-to-noise ratio as low as − 4 dB, it still maintains diagnostic accuracies of 94.2%, 93.5%, and 95.8%, respectively. Ablation studies confirm the synergistic enhancement effect among the modules: the multi-view mechanism drives high precision, while cross-attention drives high recall, and their collaboration achieves an optimal balance.

## Introduction

### Research background and motivation

As key components in high-end equipment such as wind turbines and high-speed trains, rolling bearings have a health status that is closely related to the operational safety and reliability of the entire mechanical system^1^. However, in real-world industrial scenarios, bearings often operate under harsh conditions characterized by variable operating conditions and strong noise^2^. The weak impulse signals induced by early-stage faults are easily overwhelmed by strong background noise, resulting in disordered temporal waveforms and blurred frequency spectra. Although traditional signal processing methods—such as wavelet transform^3^ and empirical mode decomposition^4^—have been widely applied, they rely heavily on manual parameter tuning and prior knowledge, making them difficult to adapt to the automated processing of massive data^5^. To overcome this bottleneck, data-driven methods, particularly deep learning, have emerged. Convolutional neural networks (CNNs)^6^, long short-term memory (LSTM) networks^7^ and their derived bidirectional architectures^8,9^ , as well as hybrid dual-stream models^10^, leverage powerful adaptive feature extraction capabilities and have demonstrated diagnostic accuracy far surpassing traditional algorithms under standard operating conditions, showing great potential for application.

Nevertheless, existing deep learning methods still face two key bottlenecks when dealing with strong noise and complex spatiotemporal dynamic signals:

First, the fragility of manifold topology under strong noise.Standard CNNs and RNNs treat vibration signals as regular Euclidean grid data and fail to capture the intrinsic manifold geometry of nonlinear dynamical systems^11^. Although graph neural networks (GNNs) provide a pathway for modeling topological relationships, most existing methods^12–14^ rely on static or single-view graph construction strategies. Under strong noise, such strategies are highly susceptible to Euclidean distance distortion, leading to spurious connections and the collapse of graph topology^15^. Multi-view learning, which enhances data representation by fusing complementary information from multiple sources, has achieved significant success in fields such as clustering and classification^16,17^ and has developed into a systematic theoretical framework^18^. The complementarity principle revealed by multi-view learning offers important insights for noise-robust feature learning. However, how to organically integrate multi-view concepts with manifold geometric reconstruction to counteract noise-induced topology collapse remains systematically unexplored in bearing fault diagnosis.

Second, insufficient depth of fusion of heterogeneous spatiotemporal features. To take advantage of complementary spatiotemporal information, recent studies have begun exploring dual-stream architectures^19,20^. However, most existing methods employ shallow fusion strategies such as simple feature concatenation or scalar attention weighting, neglecting the deep semantic alignment required between spatiotemporal heterogeneous features, thereby limiting the model’s ability to decouple complex compound faults. Although spatiotemporal joint modeling has matured in areas such as skeleton-based action recognition^21^, trajectory prediction^22^, and video inpainting^23^, the inherent non-stationarity, extremely low signal-to-noise ratio, and dynamically degraded manifold topology of bearing vibration signals preclude the direct transfer of existing methods. Moreover, explorations in other domains on enhancing noise robustness through adaptive structural design^24^ provide useful references for this work.

Based on the above analysis, a key challenge that urgently needs to be addressed is how to construct an end-to-end diagnostic framework capable of adaptively reconstructing the intrinsic manifold geometry of signals under strong noise while achieving deep semantic interaction between heterogeneous spatiotemporal features.

### Main contributions

To address the above two bottlenecks, this paper proposes a Multi-view Dynamic Manifold Reconstruction and Adaptive Cross-Attention Fusion Network (MDM-CA Net). The main contributions are as follows:

1. A multi-view dynamic manifold reconstruction mechanism is proposed. Unlike the static or single-view graph construction strategies commonly adopted in existing methods^12–14^ , this paper constructs dynamic K-nearest neighbor graphs in both the time domain and the frequency domain based on the theory of phase space reconstruction, and extracts manifold features via a dual-channel graph convolutional network. Inspired by the complementarity principle in multi-view learning^16,17^ , this mechanism leverages the sensitivity of the time-domain view to transient impacts and the sensitivity of the frequency-domain view to periodic components for complementary fusion. It achieves structural isolation of noise outliers starting from the graph construction stage, fundamentally enhancing the noise robustness of graph convolution operations.
2. An adaptive cross-attention fusion strategy is designed. Different from the shallow concatenation or self-attention fusion methods used in existing dual-stream networks^19,20,25^, this paper introduces a Transformer-style cross-attention module that explicitly establishes a cross-modal retrieval mechanism between temporal evolution features and spatial topological features. This enables nonlinear alignment and dynamic interaction of heterogeneous spatiotemporal features in a deep semantic space, significantly improving the model’s capability to decouple complex compound faults.
3. An end-to-end noise-robust diagnostic framework is constructed and validated. Extensive experiments are conducted on three public benchmark datasets, CWRU, SEU, and PU, covering same-domain, cross-domain, and various noise-type scenarios. The results demonstrate that MDM-CA Net outperforms existing state-of-the-art models in both diagnostic accuracy and stability, with particularly significant advantages under extreme conditions where the signal-to-noise ratio is as low as -4 dB. Ablation studies further reveal the synergistic enhancement effect among the modules: the multi-view mechanism drives high precision, while cross-attention drives high recall, and their collaboration achieves an optimal balance.

It should be emphasized that dynamic K-nearest neighbor graphs, graph convolutional networks, and cross-attention mechanisms are all mature techniques in their respective fields. The core innovation of this paper does not lie in any single technique, but rather in proposing a theoretically grounded and synergistically complementary systematic solution to two specific bottlenecks in bearing fault diagnosis—manifold topology collapse induced by strong noise and shallow fusion of heterogeneous spatiotemporal features.

## Related work

### Deep learning for fault diagnosis

In recent years, data-driven methods, particularly deep learning, have received widespread attention in the field of rotating machinery fault diagnosis, gradually replacing traditional signal processing methods that rely on manual feature engineering. Current research primarily revolves around two types of models: convolutional neural networks (CNNs) and recurrent neural networks (RNNs)^26^. CNN-based methods, such as WDCNN^27^ , leverage adaptive convolutional kernels to extract translation-invariant local features from raw vibration signals or time-frequency representations, achieving high diagnostic accuracy under standard operating conditions. Meanwhile, RNNs and their variants—such as long short-term memory (LSTM) and gated recurrent units (GRUs)^28^—focus on modeling the dynamic evolution relationships within time series to capture the long-range dependencies of fault signals. To further enhance temporal modeling capability, bidirectional architectures (e.g., BiTCN^8^ , BiLSTM^9^) have been introduced to exploit both forward and backward contextual information.

Building on this foundation, several studies have attempted to hybridize CNNs and RNNs to enhance the representation capability for complex signals. For instance, the DSCNN-BiGRU architecture^10^ employs dual-scale convolutions to extract multi-resolution local features and utilizes BiGRU to model temporal dependencies, achieving improved noise robustness to some extent. These hybrid architectures indicate that jointly modeling local structures and temporal evolution helps alleviate the representational limitations of single-stream networks. However, in real-world industrial environments, strong background noise and operating condition fluctuations often interfere with the feature learning process, causing models to extract redundant, fault-irrelevant information. To address this, researchers have explored noise suppression strategies at the feature level. The deep residual shrinkage network (DRSN)^29^ embeds a soft thresholding module within the residual structure to adaptively suppress noise-related feature components. Additionally, for domain shift issues, unsupervised domain adaptation methods such as domain-adversarial neural networks (DANN)^30^ and correlation alignment (CORAL)^31^ minimize the distribution discrepancy between the source and target domains to improve cross-domain generalization, offering alternative solutions for fault diagnosis under varying operating conditions.

Although the above methods have shown promising results in their respective scenarios, they are generally based on the assumption of Euclidean feature spaces, treating vibration signals as regular time series or grid-like representations. This assumption overlooks a key fact: vibration signals generated by nonlinear dynamical systems often lie on low-dimensional manifolds embedded in high-dimensional spaces. When strong noise distorts the geometric relationships in the feature space, methods relying solely on convolution, recurrence, or thresholding struggle to maintain the stability of structural features, thereby limiting their generalization capability under extremely low signal-to-noise ratios and cross-domain operating conditions.

### Graph neural networks in signal processing

Graph neural networks (GNNs) are a class of deep learning models specifically designed for graph-structured data, capable of mining topological relationships among nodes. Among them, graph convolutional networks (GCNs)^32^ are particularly effective at extracting spatial features from graph topologies. Existing GCN-based fault diagnosis methods can be categorized into the following two classes according to their graph construction strategies:

1. Static construction based on frequency-domain topology. Researchers often transform one-dimensional vibration signals into frequency-domain or time-frequency representations as the basis for graph construction. For example, Wei et al.^2^ used fast Fourier transform (FFT) to convert signals into the frequency domain and then constructed fully connected graphs for GCN processing, achieving better noise robustness than traditional CNNs. Yan et al.^14^ employed constant-Q transform (CQT) to generate spectrograms and defined the adjacency matrix based on harmonic relationships among frequency bins. Wang et al.^33^ proposed ERW-GCN, introducing an equal-ratio weighted adjacency matrix combined with principal component analysis (PCA) for dimensionality reduction and graph weighting. Although these methods exploit the inherent sparsity of the frequency domain, the graph structures they adopt are typically static or predefined–once the adjacency matrix is constructed, it remains unchanged during feature propagation, failing to adapt to dynamic topological variations caused by fault evolution. Although Yin et al.^34^ attempted to alleviate this limitation by constructing multi-scale graphs using horizontal visibility graphs (HVG), single-view graph representations still tend to lose transient impact information from the time domain, restricting the ability to capture non-stationary fault features.
2. Adaptive construction based on temporal proximity. To capture richer dynamic information, other methods construct graphs directly in the time domain or in reconstructed phase spaces. A common strategy is to use the K-nearest neighbor (K-NN) algorithm to dynamically identify relevant neighbors. Zuo et al.^35^ explored converting time series into graphs using visibility graphs (VG) to preserve global structural information. However, time-domain construction mechanisms such as K-NN or VG are highly sensitive to noise: under low signal-to-noise ratio conditions, Gaussian white noise severely distorts Euclidean distances, causing noisy outliers to be incorrectly connected and generating ”spurious connections,” thereby destroying the integrity of the manifold structure.

Both of the above types of methods almost exclusively adopt single-view strategies, failing to simultaneously account for the impact sensitivity of the time domain and the periodic sparsity of the frequency domain. Consequently, they cannot fully reconstruct the underlying manifold of the signal. From the theoretical perspective of multi-view learning, a single view can only capture one facet of the data, whereas complementary fusion of multiple views significantly enhances the completeness and robustness of representations^16,17^. Recent studies have further confirmed the value of multi-view strategies in graph structure optimization—for example, the anchor-to-graph structure collaborative regularization method for multi-view clustering (AGSCR-MVC) proposed by Guo et al.^36^ achieves joint optimization of clustering structure and discriminative capability by synergistically exploring the structural semantics of anchors and anchor graphs, demonstrating the great potential of multi-view complementary information in graph learning.

### Spatio-temporal interaction modeling

Spatiotemporal joint modeling aims to simultaneously capture the spatial structural information and temporal evolution patterns of data, and has become a core technique in multiple research fields. Yan et al.^21^ proposed the spatiotemporal graph convolutional network (ST-GCN) for skeleton-based action recognition, which models skeleton sequences as spatiotemporal graphs and aggregates both spatial neighborhood and temporal neighborhood information via graph convolution. Their spatial configuration partitioning strategy effectively captures the directional characteristics of joint movements. Selvaraj et al.^22^ proposed TrajectoFormer for autonomous driving trajectory prediction, which employs eight-neighborhood spatiotemporal modeling and a dual-branch Transformer encoder-decoder architecture, significantly improving the ability to model complex spatiotemporal interaction dependencies among vehicles. Zhang et al.^23^ proposed ESTINet for video inpainting, adopting a three-stage architecture (spatial feature extraction $\rightarrow$ spatiotemporal interaction learning $\rightarrow$ spatiotemporal consistency enhancement). By using an improved LSTM structure, this method achieves efficient modeling of temporal correlations with extremely low computational overhead, striking an excellent balance between speed and performance.

The above works demonstrate the powerful capability of spatiotemporal interaction modeling. However, it should be noted that the data processed by these methods are fundamentally different from bearing vibration signals. Skeleton data have clear joint topologies and relatively high signal-to-noise ratios; trajectory data exhibit regular spatiotemporal evolution patterns; and video frames contain rich spatial texture information. In contrast, bearing vibration signals have three notable peculiarities: (1) Non-stationarity—fault impacts appear as transient and irregular features in the time domain; (2) Extremely low signal-to-noise ratio—weak fault pulses are often submerged by strong background noise; (3) Dynamic degradation of manifold topology—noise distorts geometric distances in the phase space, leading to the collapse of graph structures. These peculiarities make it difficult to achieve satisfactory results by directly transferring existing spatiotemporal interaction frameworks. Therefore, based on a spatiotemporal dual-stream architecture, this paper specifically designs a multi-view dynamic manifold reconstruction mechanism to address the noise-induced degradation of spatial topology, and achieves deep semantic alignment of heterogeneous spatiotemporal features via cross-attention (rather than self-attention or simple concatenation).

### Attention mechanism and feature fusion

While optimizing the spatial topological representation, comprehensive fault diagnosis also requires simultaneous consideration of both spatial topology and temporal evolution. Consequently, parallel “dual-stream” architectures consisting of a GCN branch (responsible for topological features) and an RNN/CNN branch (responsible for evolutionary features) have gradually become a mainstream choice, such as DS-STFN^37^. Within this framework, how to effectively fuse heterogeneous features originating from different domains (time domain vs. spatial domain) becomes a primary challenge.

In recent years, attention mechanisms have been widely adopted for feature enhancement and fusion. Singh et al.^38^ combined graph attention networks (GAT) with LSTM to enhance graph representation capabilities at the node level; Zhang et al.^39^ proposed a multi-head multi-sensor graph attention network (MMHGAT) to integrate the contributions of different sensors. However, these methods mainly focus on enhancing feature representation within a single modality and do not address cross-modal interaction between heterogeneous spatiotemporal features.

With the success of the Transformer architecture in natural language processing and computer vision, self-attention mechanisms have been gradually introduced into the field of rotating machinery fault diagnosis to capture long-range dependencies. CNN-Transformer^25^ demonstrated the significant advantages of self-attention in capturing global correlations, effectively compensating for the limited receptive field of traditional convolutional networks. Nevertheless, most dual-stream fusion models, including CNN-Transformer, adopt either shallow concatenation or single-stream self-attention—where attention computation is typically confined within the same feature space. Although such designs can enhance the global modeling capability of features, they struggle to explicitly characterize deep semantic interactions between heterogeneous features.

In contrast, the cross-attention mechanism^40^ provides a more expressive fusion paradigm. Unlike self-attention, which only reorganizes information within a single modality, cross-attention allows one modality to serve as the query to actively retrieve key key-value pairs from another modality, thereby achieving cross-modal information alignment and dynamic reorganization. Although cross-attention has demonstrated powerful feature alignment capabilities in the field of multimodal learning, research in bearing fault diagnosis remains relatively scarce–particularly regarding the establishment of deep semantic interactions between multi-view manifold features and temporal evolution features, where no systematic work has yet been reported. This is precisely the innovation of this paper: by using temporal evolution features as queries to retrieve critical topological patterns from the spatial manifold, ”on-demand” cross-modal information acquisition is achieved, thereby effectively decoupling compound fault features under strong noise and complex operating conditions.

## Preliminaries

To address the issue mentioned above—that traditional deep learning models struggle to process non-Euclidean spatial data—this chapter first introduces the theoretical foundations.

### Graph convolutional networks

Graph convolutional networks (GCNs)^32^ are a class of deep learning models adept at processing non-Euclidean structural data, such as manifold graphs $\mathcal {G}$. GCNs can be employed to extract spatial topological features from constructed dynamic manifold graphs. Given an undirected graph $\mathcal {G}=(\mathcal {V}, \mathcal {E})$, where $\mathcal {V}$ is the vertex set containing *N* nodes and $\mathcal {E}$ is the edge set, the graph structure is described by an adjacency matrix $\textbf{A} \in \mathbb {R}^{N \times N}$, where $\textbf{A}_{ij}=1$ indicates a connection between node *i* and node *j*, and 0 otherwise. To prevent numerical instability during multi-layer feature propagation, a renormalization trick is commonly applied. The layer-wise propagation rule of a GCN can be expressed as:

1 $$\begin{aligned} \textbf{H}^{(l+1)}=\sigma \left( \widetilde{\textbf{D}}^{-\frac{1}{2}} \widetilde{\textbf{A}} \widetilde{\textbf{D}}^{-\frac{1}{2}} \textbf{H}^{(l)} \textbf{W}^{(l)}\right) \end{aligned}$$

where $\textbf{H}^{(0)}$ is the initial node feature matrix $\textbf{X}_{\text {node}}$, $\widetilde{\textbf{A}}=\textbf{A}+\textbf{I}_N$ is the adjacency matrix with added self-loops, $\textbf{I}_N$ is the identity matrix, and $\widetilde{\textbf{D}}$ is the degree matrix of $\widetilde{\textbf{A}}$ with diagonal entries $\widetilde{D}_{ii}=\sum _j \tilde{A}_{ij}$. $\textbf{H}^{(l)} \in \mathbb {R}^{N \times d_l}$ is the input feature matrix of the *l*-th layer, $\textbf{W}^{(l)}$ is the trainable weight matrix of the current layer, and $\sigma (\cdot )$ is a nonlinear activation function, which is ReLU in this paper.

### Bidirectional gated recurrent unit

To capture the dynamic evolution of vibration signals along the time dimension, bidirectional gated recurrent units (BiGRUs) can be employed^28,41^. Compared with LSTMs, GRUs have a simpler gating structure and typically involve fewer parameters. By modeling information bidirectionally and fusing both forward and backward contexts, BiGRUs often achieve better representation performance in offline sequence modeling. A BiGRU consists of a forward GRU and a backward GRU, which read the input sequence $\textbf{X}_{seq}$ in the forward and reverse directions, respectively. For the input $\textbf{x}_t$ at the *t*-th time step in the sequence, the forward hidden state $\overrightarrow{\textbf{h}_t}$ and the backward hidden state $\overleftarrow{\textbf{h}_t}$ are computed as follows:

2 $$\begin{aligned} \begin{aligned} \overrightarrow{\textbf{h}_t}&=\operatorname {GRU}\left( \overrightarrow{\textbf{h}_{t-1}}, x_t\right) \\ \overleftarrow{\textbf{h}}_t&=\operatorname {GRU}\left( \overleftarrow{\textbf{h}_{t+1}}, x_t\right) \end{aligned} \end{aligned}$$

The final output $\textbf{h}_t$ of the BiGRU at time *t* is the concatenation of the hidden states from both directions:

3 $$\begin{aligned} \textbf{h}_t=\left[ \overrightarrow{\textbf{h}_t} \oplus \overleftarrow{\textbf{h}_t}\right] \in \mathbb {R}^{2 d_h} \end{aligned}$$

This bidirectional mechanism ensures that the model can utilize both past and future contextual information at each time step *t*, thereby providing a more comprehensive representation of the temporal dependencies in the signal.

### Scaled dot-product attention

The core idea of the attention mechanism^40^ is to enable a model to adaptively focus on the critical parts of the input information according to the requirements of the current task. This study adopts the standard scaled dot-product attention as the computational core. Given a query matrix $\textbf{Q} \in \mathbb {R}^{T \times d_k}$, a key matrix $\textbf{K} \in \mathbb {R}^{N \times d_k}$, and a value matrix $\textbf{V} \in \mathbb {R}^{N \times d_v}$, the attention scores are computed as:

4 $$\begin{aligned} \operatorname {Attention}(\textbf{Q}, \textbf{K}, \textbf{V})=\operatorname {softmax}\left( \frac{\textbf{Q K}^T}{\sqrt{d_k}}\right) \textbf{V} \end{aligned}$$

Here, the dot product $\textbf{Q K}^T$ computes the similarity between the queries and all keys; $\sqrt{d_k}$ is a scaling factor that prevents the dot product from becoming too large, which could cause vanishing gradients in the Softmax function. The Softmax function normalizes the similarities into probabilistic weights, which are then used to compute a weighted sum with $\textbf{V}$.

## Proposed method

### Overall architecture

**Fig. 1:**
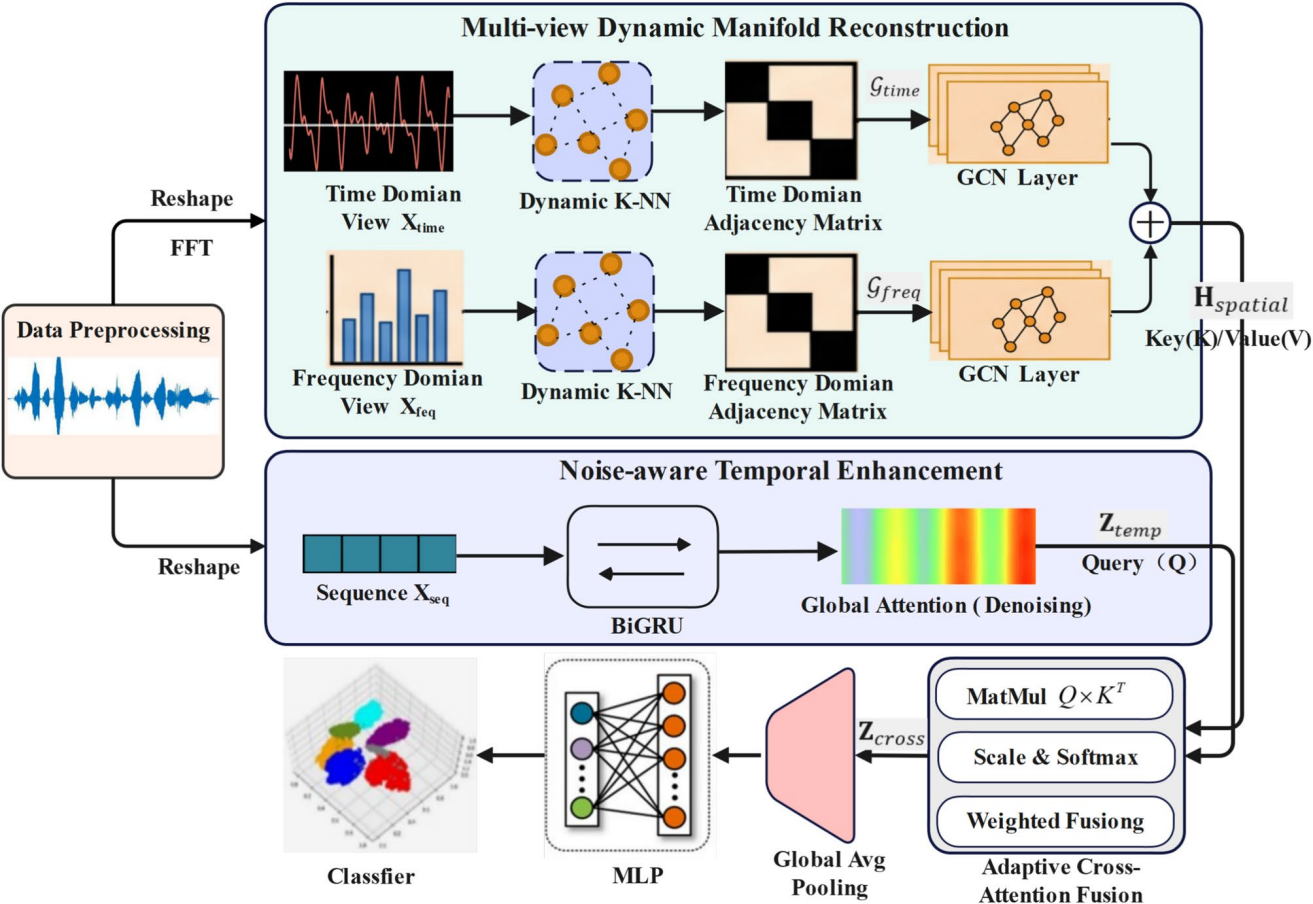
MDM-CA net architecture.

The overall architecture of MDM-CA Net is shown in Fig. 1, which consists of three core modules: the spatial stream, the temporal stream, and the adaptive cross-attention fusion module. The overall workflow is as follows:

1. Multi-domain signal preprocessing. First, the input raw vibration signal sample $\textbf{x} \in \mathbb {R}^L$ is mapped into both the time domain and the frequency domain. The time-domain branch preserves the original sequence structure to retain transient impact features, while the frequency-domain branch obtains spectral information via fast Fourier transform to capture periodic fault characteristics. The time-domain and frequency-domain features are jointly used for manifold reconstruction in the spatial stream, whereas the time-domain sequence is fed into the temporal stream for evolution modeling.
2. Spatial stream: multi-view dynamic manifold reconstruction. This module aims to mine the non-Euclidean geometric structure of the signal hidden in the phase space. Unlike traditional single-view graph construction, we construct dynamic K-nearest neighbor graphs in both the time domain and the frequency domain. Through a dual-channel graph convolutional network (GCN), the model aggregates topological neighborhood information from different views, effectively filtering out spurious connections caused by random noise and extracting robust spatial manifold features.
3. Temporal stream: noise-aware temporal enhancement. The temporal stream focuses on modeling the temporal evolution patterns of the signal. A bidirectional gated recurrent unit (BiGRU) is employed to capture long-range dependencies in the vibration sequence. Furthermore, to further suppress background noise, a global attention mechanism is introduced at the output of the BiGRU, which adaptively assigns high weights to key time steps containing fault impacts, thereby generating denoised temporal evolution features.
4. Adaptive cross-attention fusion. To achieve deep interaction between heterogeneous features, the model designs a Transformer-based cross-attention module. Instead of simple feature concatenation, this module uses the temporal stream features as queries to retrieve key key-value pairs from the spatial stream features, enabling semantic-level automatic alignment and weighted fusion of spatiotemporal features.

Finally, the fused feature vector is fed into a fully connected layer and a Softmax classifier to output the probability distribution of the bearing health states. Through this synergistic design of multiple views and multiple streams, MDM-CA Net achieves high-precision fault diagnosis under strong noise and variable operating conditions.

### Spatial stream: multi-view dynamic manifold reconstruction

To mine the intrinsic topological structure from non-stationary signals, this module constructs dynamic K-nearest neighbor graphs in both the time domain and the frequency domain based on the theory of phase space reconstruction^42^, and extracts noise-resilient manifold features via a graph convolutional network.

#### Multi-view feature mapping

According to Takens’ embedding theorem^42^, a one-dimensional time series can be mapped into a high-dimensional phase space via a sliding window to recover its dynamical characteristics. Given an input raw vibration signal sample $\textbf{x} \in \mathbb {R}^L$:

- Time-domain view. $\textbf{x}$ is reshaped into a matrix $\textbf{X}_{\text {time}} \in \mathbb {R}^{N \times d_{in}}$, where *N* is the number of nodes and $d_{in}$ is the feature dimension per node ($L = N \times d_{in}$). Each row vector represents a local segment of the signal and is regarded as a node in the graph.

- Frequency-domain view. Fast Fourier transform (FFT) is performed on $\textbf{x}$, and the magnitude spectrum is taken and reshaped into $\textbf{X}_{\text {freq}} \in \mathbb {R}^{N \times d_{in}}$. This view explicitly encodes the periodic components of the signal.

These two matrices, $\textbf{X}_{\text {time}}$ and $\textbf{X}_{\text {freq}}$, serve as the initial node features $\textbf{H}^{(0)}$ for two subsequent independent graph networks, respectively.

#### Dual-channel dynamic graph construction

To dynamically capture the local geometric structure of the signal as faults evolve, the K-nearest neighbor algorithm is employed to construct an instant-specific adjacency graph for each sample. For the feature matrix $\textbf{X}$ of a given view, the Euclidean distance between node *i* and node *j* is computed as $d_{ij} = \Vert \textbf{x}_i - \textbf{x}_j\Vert _2$ (where $\textbf{x}_i$ is the *i*-th row vector of $\textbf{X}$). For each node *i*, only the *K* nearest neighbors are retained to construct a sparse adjacency matrix $\textbf{A} \in \mathbb {R}^{N \times N}$:

5 $$\begin{aligned} A_{ij}=\left\{ \begin{array}{cc} \exp \left( -\frac{d_{ij}^2}{\sigma ^2}\right) , & \text {if } j \in \mathcal {N}_K(i) \\ 0, & \text {otherwise} \end{array}\right. \end{aligned}$$

where $\mathcal {N}_K(i)$ denotes the set of *K* nearest neighbors of node *i*, and $\sigma$ is a hyperparameter controlling the kernel width. Consequently, the time-domain adjacency matrix $\textbf{A}_{\text {time}}$ and the frequency-domain adjacency matrix $\textbf{A}_{\text {freq}}$ are obtained, respectively.

This dynamic graph construction strategy adaptively connects signal segments with similar patterns. Under strong noise interference, noise points typically appear as random outliers and are unlikely to enter the K-nearest neighbor set, thereby being physically isolated at the graph construction stage, achieving structural denoising at the source. The theoretical robustness analysis of this mechanism under strong noise conditions is provided in the Supplementary Information.

#### Manifold feature extraction via GCN

After constructing the time-domain graph $\mathcal {G}_{\text {time}} = (\textbf{A}_{\text {time}}, \textbf{X}_{\text {time}})$ and the frequency-domain graph $\mathcal {G}_{\text {freq}} = (\textbf{A}_{\text {freq}}, \textbf{X}_{\text {freq}})$, the GCN layers defined by the graph convolutional network are used to perform feature aggregation. Let the output of the *l*-th layer be $\textbf{H}^{(l)}$. The propagation process of the dual channels is then given by:

6 $$\begin{aligned} \begin{aligned} \textrm{H}_{\text{ time } }^{(l+1)}&=\operatorname {ReLU}\left( \widetilde{\textrm{D}}_{\text{ time } }^{-\frac{1}{2}} \widetilde{\mathrm {~A}}_{\text{ time } } \widetilde{\textrm{D}}_{\text{ time } }^{-\frac{1}{2}} \textrm{H}_{\text{ time } }^{(l)} \textrm{W}_{\text{ time } }^{(l)}\right) \\ \textrm{H}_{\text{ freq } }^{(l+1)}&=\operatorname {ReLU}\left( \widetilde{\textrm{D}}_{\text{ freq } }^{-\frac{1}{2}} \widetilde{\mathrm {~A}}_{\text{ freq } } \widetilde{\textrm{D}}_{\text{ freq } }^{-\frac{1}{2}} \textrm{H}_{\text{ freq } }^{(l)} \textrm{W}_{\text{ freq } }^{(l)}\right) \end{aligned} \end{aligned}$$

where $\widetilde{\textbf{A}} = \textbf{A} + \textbf{I}$ is the adjacency matrix with added self-loops, and $\widetilde{\textbf{D}}$ is the corresponding degree matrix.

After *L* layers of convolution, the output features from the two views are adaptively fused in a weighted manner to obtain the final spatial stream feature $\textbf{H}_{\text {spatial}} \in \mathbb {R}^{N \times d_{\text {out}}}$:

7 $$\begin{aligned} \textbf{H}_{\text{ spatial } }=\textbf{H}_{\text{ time } }^{(L)}+\lambda \cdot \textbf{H}_{\text{ freq } }^{(L)} \end{aligned}$$

where $\lambda$ is a learnable balancing coefficient that automatically adjusts the importance of the time-domain and frequency-domain views. This feature matrix $\textbf{H}_{\text {spatial}}$ will serve as the Key and Value in the subsequent cross-attention module.

### Temporal stream: noise-aware temporal enhancement

To capture the dynamic evolution characteristics of fault signals over time and suppress transient noise interference, this module constructs a temporal feature extractor centered on a BiGRU and integrates a global attention mechanism to achieve adaptive denoising.

#### BiGRU temporal dependency modeling

The raw one-dimensional vibration signal $\textbf{x} \in \mathbb {R}^L$ is reshaped into a time series $\textbf{X}_{seq} \in \mathbb {R}^{T \times d_{in}}$, where *T* is the number of time steps. A BiGRU layer is employed to capture both forward and backward temporal dependencies of the sequence. Let the input at the *t*-th time step be $\textbf{x}_t$; the hidden state update process of the BiGRU is then expressed as:

8 $$\begin{aligned} \textbf{h}_t=\operatorname {BiGRU}\left( \textbf{x}_t, \textbf{h}_{t-1}\right) =\left[ \overrightarrow{\textbf{h}_t} \oplus \overleftarrow{\textbf{h}_t}\right] \in \mathbb {R}^{2 d_h} \end{aligned}$$

After stacking $L_{gru}$ layers, a temporal feature matrix containing rich contextual information, $\textbf{H}_{seq} = [\textbf{h}_1, \textbf{h}_2, \ldots , \textbf{h}_T]^T \in \mathbb {R}^{T \times 2 d_h}$, is obtained. This matrix encodes not only the instantaneous features at each time step but also the global temporal evolution trends.

#### Global attention denoising mechanism

Given that not all time steps contain valid fault information under strong noise environments, directly using $\textbf{H}_{seq}$ may introduce noise features. To address this, a global attention mechanism is introduced at the output of the BiGRU to adaptively assign importance weights to each time step. For each hidden state $\textbf{h}_t$, the attention weight $\alpha _t$ is computed as follows:

9 $$\begin{aligned} \textbf{u}_t = \tanh (\textbf{W}_w \textbf{h}_t + \textbf{b}_w) \end{aligned}$$

10 $$\begin{aligned} \alpha _t = \frac{\exp (\textbf{u}_t^T \textbf{u}_s)}{\sum _{t=1}^T \exp (\textbf{u}_t^T \textbf{u}_s)} \end{aligned}$$

where $\textbf{W}_w$ and $\textbf{b}_w$ are learnable projection matrix and bias, respectively, and $\textbf{u}_s$ is a randomly initialized context vector that measures the relevance of each time step to fault patterns.

Finally, the denoised temporal feature matrix $\textbf{Z}_{\text {temp}} \in \mathbb {R}^{T \times 2d_h}$ is obtained through element-wise weighting:

11 $$\begin{aligned} \textbf{Z}_{\text {temp}} = [\alpha _1 \textbf{h}_1, \alpha _2 \textbf{h}_2, \ldots , \alpha _T \textbf{h}_T]^T \end{aligned}$$

The attention weights $\alpha _t$ adaptively capture and enhance the critical segments containing fault impacts (i.e., regions with high signal-to-noise ratios) while suppressing irrelevant time steps dominated by background noise. This feature matrix $\textbf{Z}_{\text {temp}}$ will serve as the Query in the subsequent cross-attention module, guiding the model to retrieve relevant topological structures from the spatial stream.

### Adaptive cross-attention fusion module

To overcome the limitations of shallow feature concatenation or simple gating mechanisms in traditional dual-stream networks, this module introduces a Transformer-based cross-attention mechanism. This allows the temporal features to actively serve as queries to retrieve the key key-value pairs from the spatial stream features, thereby achieving nonlinear interaction and alignment of heterogeneous features in a deep semantic space.

#### Query-key-value mapping

First, the denoised temporal features $\textbf{Z}_{\text {temp}} \in \mathbb {R}^{T \times 2d_h}$ and the spatial manifold features $\textbf{H}_{\text {spatial}} \in \mathbb {R}^{N \times d_{\text {out}}}$ are respectively projected into a latent space of the same dimension $d_k$. The specific linear transformations are as follows:

12 $$\begin{aligned} \begin{aligned} \textbf{Q}&= \textbf{Z}_{\text {temp}} \textbf{W}_q \\ \textbf{K}&= \textbf{H}_{\text {spatial}} \textbf{W}_k \\ \textbf{V}&= \textbf{H}_{\text {spatial}} \textbf{W}_v \end{aligned} \end{aligned}$$

where $\textbf{W}_q \in \mathbb {R}^{2d_h \times d_k}$, $\textbf{W}_k \in \mathbb {R}^{d_{\text {out}} \times d_k}$, and $\textbf{W}_v \in \mathbb {R}^{d_{\text {out}} \times d_v}$ are learnable projection matrices. Here, $\textbf{Q}$ represents the temporal evolution patterns of the fault, while $\textbf{K}$ and $\textbf{V}$ represent the indices and contents of the spatial topological structure, respectively. Through this asymmetric mapping, the model explicitly establishes a retrieval mechanism indexed by “temporal evolution” and content-based on “spatial structure”.

#### Deep feature interaction computation

Using the scaled dot-product attention formula, the correlation score matrix $\textbf{S}_{\text {attn}} \in \mathbb {R}^{T \times N}$ between the temporal query $\textbf{Q}$ and the spatial key $\textbf{K}$ is computed as follows:

13 $$\begin{aligned} \textbf{S}_{attn} = \operatorname {softmax}\left( \frac{\textbf{Q}\textbf{K}^T}{\sqrt{d_k}}\right) \end{aligned}$$

Here, each element $S_{ti}$ of the matrix quantifies the adaptive attention weight that the transient evolution feature at the *t*-th time step assigns to the topological information of the *i*-th spatial node. This weight reflects the degree of alignment between heterogeneous features in a deep semantic space. Subsequently, this matrix is used to perform a weighted aggregation of the spatial values $\textbf{V}$, achieving a deep fusion of spatiotemporal features and yielding the fused contextual feature $\textbf{Z}_{\text {cross}} \in \mathbb {R}^{T \times d_v}$:

14 $$\begin{aligned} \textbf{Z}_{\text {cross}} = \textbf{S}_{\text {attn}} \textbf{V} \end{aligned}$$

For the final fault classification, global average pooling is applied to $\textbf{Z}_{\text {cross}}$ to compress redundant information along the temporal dimension, generating a compact feature vector. This vector is then fed into a multi-layer perceptron (MLP) and a Softmax classifier to output the probability distribution over fault categories, $\textbf{P} \in \mathbb {R}^C$:

15 $$\begin{aligned} \textbf{P} = \operatorname {softmax}\left( \operatorname {MLP}\left( \frac{1}{T} \sum _{t=1}^T \textbf{z}_{\text {cross}, t}\right) \right) \end{aligned}$$

This query-based cross-retrieval mechanism endows the model with an on-demand information acquisition capability: the model can dynamically extract the most relevant feature clues from the global manifold topology according to the current dynamical evolution state, thereby significantly enhancing its feature decoupling ability and diagnostic robustness in complex compound fault scenarios.

## Experimental analysis

### Experimental setup

#### Datasets and preprocessing

Three publicly available bearing fault datasets are selected for experimental validation in this section: the CWRU Bearing Dataset, the SEU Gearbox Dataset, and the PU Bearing Dataset. These three datasets differ in fault types, acquisition conditions, and noise characteristics, enabling a comprehensive evaluation of the diagnostic performance and generalization capability of the proposed method from multiple perspectives.

1. Dataset description

- CWRU bearing dataset

The CWRU dataset is provided by the Bearing Data Center of Case Western Reserve University and is one of the most widely used benchmark datasets in the field of bearing fault diagnosis. The test rig consists of a 2 horsepower (HP) motor, a torque sensor, and a power tester. Vibration signals are collected by an accelerometer mounted on the motor drive end (DE) at a sampling frequency of 12 kHz. The dataset includes a normal condition as well as three fault types: inner race fault (IF), outer race fault (OF), and ball fault (BF). Each fault type contains three damage diameters of 0.007, 0.014, and 0.021 inches, resulting in a total of 10 health states. In this study, data under the 0 HP load condition are selected for analysis. The detailed sample distribution is shown in Table 1.

**Table 1:** Sample distribution of the CWRU dataset.

Fault type	Damage diameter (in)	Category label	Training samples	Testing samples
Normal	–	0	800	200
Inner race fault (IF)	0.007	1	800	200
Inner race fault (IF)	0.014	2	800	200
Inner race fault (IF)	0.021	3	800	200
Ball fault (BF)	0.007	4	800	200
Ball fault (BF)	0.014	5	800	200
Ball fault (BF)	0.021	6	800	200
Outer race fault (OF)	0.007	7	800	200
Outer race fault (OF)	0.014	8	800	200
Outer race fault (OF)	0.021	9	800	200

- Southeast University (SEU) gearbox dataset

The SEU dataset is provided by the School of Mechanical Engineering at Southeast University and was collected from a planetary gearbox test rig. This dataset contains fault signals from both bearings and gears; this study uses the bearing fault subset. The vibration signals were sampled at a frequency of 20 kHz, and experiments were conducted under two operating conditions: Condition 1 (rotational speed 20 Hz, load 0 V) and Condition 2 (rotational speed 30 Hz, load 2 V). The dataset includes five bearing health states: Normal (N), inner race fault (IF), outer race fault (OF), ball fault (BF), and a compound fault of both inner and outer races (IF+OF). In this study, data from Condition 1 are used for training, while data from Condition 2 are used for testing, in order to evaluate the cross-domain generalization capability of the model. The detailed sample distribution is shown in Table 2.

**Table 2:** Sample distribution of the SEU dataset.

Fault type	Category label	Training samples (condition 1)	Testing samples (condition 2)
Normal (N)	0	800	200
Inner race fault (IF)	1	800	200
Outer race fault (OF)	2	800	200
Ball fault (BF)	3	800	200
Compound fault (IF+OF)	4	800	200

- Paderborn University (PU) bearing dataset

To further validate the generalization capability of the model in scenarios closer to real industrial practice, the Paderborn University (PU) bearing dataset is introduced. Unlike the artificial damages created by electrical discharge machining (EDM) in the CWRU and SEU datasets, the PU dataset includes both artificial damages and real damages generated from accelerated lifetime tests. The latter more closely resemble the natural degradation process in actual operating conditions in terms of damage morphology and severity. The test rig consists of a permanent magnet synchronous motor, a torque measuring shaft, a rolling bearing test module, a flywheel, and a load motor. Vibration signals are collected by accelerometers mounted on the bearing housing at a sampling frequency of 64 kHz. In this study, the operating condition with a rotational speed of 1500 rpm, a load torque of 0.7 Nm, and a radial force of 1000 N is selected for analysis. Following the mainstream practice of this dataset in the fault diagnosis field, three health states are selected for classification experiments: healthy bearings (K001, K002, K003, K004, K005, K006), artificial inner race fault bearings (KI01, KI03, KI05, KI07), and artificial outer race fault bearings (KA01, KA03, KA05, KA07). The detailed sample distribution is shown in Table 3.

**Table 3:** Sample distribution of the PU dataset .

Fault type	Category label	Training samples	Testing samples
Normal (N)	0	800	200
Inner race fault (IF)	1	800	200
Outer race fault (OF)	2	800	200

(2) Data preprocessing To avoid data leakage between the training and test sets caused by overlapping sliding windows, a “split-then-segment” preprocessing strategy is adopted for all datasets.

For the CWRU and PU datasets, the original continuous vibration signal corresponding to each health state is first strictly divided into a training segment (first 80%) and a test segment (last 20%) along the time axis, ensuring no temporal overlap between the two segments. Subsequently, sliding windows are applied independently within the training and test segments to generate samples, with a window length of 1024 sampling points and an overlap rate of 75%. This procedure guarantees that all samples in the test set originate from time intervals not encountered by the training set, fundamentally eliminating cross-set information leakage due to window overlap.

For the SEU dataset, since the training and test sets come from two completely independent physical acquisition conditions–Condition 1 (rotational speed 20 Hz, load 0 V) and Condition 2 (rotational speed 30 Hz, load 2 V)–there is no risk of data leakage. Within each condition, the same sliding window strategy (window length 1024, overlap rate 75%) is applied for sample generation.

Finally, Z-score normalization is performed on all segmented samples to eliminate the influence of signal amplitude differences across different operating conditions.

#### Baselines and implementation

To comprehensively evaluate the performance of MDM-CA Net, nine representative deep learning models are selected as comparison baselines, covering five categories: basic deep learning models, graph neural network models, dual-stream fusion architectures, noise-robust models, and unsupervised domain adaptation models. They are briefly described as follows:

- Basic deep learning models

1DCNN^27^: A one-dimensional convolutional neural network with a 3-layer convolutional structure that extracts local time-domain features directly from raw vibration signals, serving as the most fundamental deep learning baseline in fault diagnosis. The kernel sizes are 64, 32, and 16, with channel numbers of 16, 32, and 64, respectively. Each convolutional layer is followed by BatchNorm, ReLU activation, and max pooling (pool size 2). The final output is obtained via global average pooling and a fully connected layer (hidden dimension 256).

BiGRU^41^: A bidirectional gated recurrent unit network that captures both forward and backward temporal dependencies of signals, used to validate the performance of pure temporal modeling methods. It adopts a 2-layer BiGRU structure with a hidden dimension of 64 (per direction). The input sequence format is consistent with the temporal stream of this paper, and the hidden state at the last time step is passed through a fully connected layer for classification.

- Graph neural network models

GCN^32^: A graph convolutional network that constructs a single-view adjacency graph based on frequency-domain features for graph convolution operations, used to compare the superiority of the multi-view graph construction strategy. It adopts a 2-layer GCN structure with a hidden dimension of 128. The adjacency graph is constructed by computing K-NN (K = 10) on the FFT magnitude spectrum features, using a static global graph construction approach (constructed once before training and kept unchanged during training and testing). After graph convolution, global average pooling and a fully connected layer are applied for classification.

GAT^38^: A graph attention network that introduces an attention mechanism to dynamically learn the importance weights of neighboring nodes during graph convolution, used to compare the performance difference between intra-graph attention and the cross-attention mechanism proposed in this paper. It adopts a 2-layer GAT structure with 4 attention heads per layer, each with a hidden dimension of 32 (concatenated to 128 dimensions). The graph construction is the same as that of the GCN baseline (static K-NN graph based on frequency-domain features, K = 10).

- Dual-stream fusion architectures

DSCNN-BiGRU^10^: A hybrid network combining dual-scale convolution and bidirectional GRU, which uses feature concatenation to fuse spatial and temporal features, used to compare the effectiveness of the cross-attention fusion strategy. The spatial branch employs dual-scale one-dimensional convolutions (kernel sizes 3 and 7, each with 2 layers, channel numbers 32 and 64). The temporal branch uses a 2-layer BiGRU (hidden dimension 64). The outputs of the two branches are directly concatenated along the feature dimension and fed into a fully connected classifier.

CNN-transformer^25^: A hybrid architecture combining a CNN and a Transformer, which uses a self-attention mechanism to model global dependencies, used to compare performance in spatiotemporal feature interaction. The CNN encoder consists of 3 layers of one-dimensional convolution (kernel size 7, channel numbers 64-128-256). The Transformer encoder has 2 layers, 4-head self-attention, a feed-forward network hidden dimension of 512, and a dropout rate of 0.1.

- Noise-robust model

DRSN^29^: A deep residual shrinkage network that adaptively suppresses noise-related features through a soft thresholding module, used to compare the robustness of the proposed model under noisy environments. It adopts 4 residual shrinkage blocks with channel numbers of 16, 32, 64, and 128, respectively. Each block embeds an adaptive soft thresholding subnetwork (learning channel-wise thresholds via global average pooling and two fully connected layers). The final output is obtained via global average pooling and a fully connected layer for classification.

- Unsupervised domain adaptation models

DANN^30^: Domain-adversarial neural network, which learns domain-invariant feature representations through adversarial training by attaching a gradient reversal layer and a domain discriminator after the feature extractor. It is one of the most classical methods in unsupervised domain adaptation. In this paper, DANN uses a 1DCNN as the backbone feature extractor to validate the performance of traditional domain adaptation methods in cross-domain bearing fault diagnosis.

CORAL^31^: Correlation alignment method, which achieves implicit domain adaptation by minimizing the difference in the second-order statistics (covariance matrices) of features between the source and target domains. CORAL also uses a 1DCNN as the backbone network, used to compare the difference between distribution alignment strategies and the dynamic manifold reconstruction strategy proposed in this paper in mitigating domain shift.

Both DANN and CORAL use a 3-layer 1DCNN as the shared feature extractor (with the same structure as the baseline 1DCNN) and a feature dimension of 256. For DANN, the domain discriminator adopts a 2-layer fully connected network (hidden dimension 128), and the adaptation factor $\lambda$ of the gradient reversal layer linearly increases from 0 to 1 according to the scheduling strategy in the original literature. For CORAL, the loss weight coefficient is set to $\lambda _{\textrm{CORAL}}=1.0$, which is added equally to the classification loss. The initial learning rate, optimizer, and training strategy of both models are kept consistent with those of the other baselines.

It should be noted that DANN and CORAL, as unsupervised domain adaptation methods, are designed to improve generalization capability under the condition of distribution mismatch between the training and test domains. Therefore, these two baselines participate only in the SEU cross-domain experiments (i.e., training on Condition 1, testing on Condition 2), where a clear domain shift exists. For the CWRU and PU datasets, both the training and test sets originate from the same operating condition without domain shift; running domain adaptation methods under such conditions lacks physical meaning and is thus not included in the comparison.

The hyperparameters of the proposed MDM-CA Net are set as follows: input sample length of 1024, batch size of 64, initial learning rate of 0.001, Adam optimizer, cosine annealing learning rate scheduling, maximum training epochs of 100, and early stopping patience of 10. Regarding network architecture, both the GCN and BiGRU adopt a 2-layer structure with hidden dimensions of 128 and 64, respectively. The number of nearest neighbors K for K-NN graph construction is 10. The cross-attention module uses 4-head attention, with a fused feature dimension of 256 and a dropout rate of 0.3.

To ensure fair comparison, all experiments are conducted under the same hardware and software environment. The experimental platform is configured as follows: Ubuntu 20.04 operating system, PyTorch 1.12.0 deep learning framework, NVIDIA RTX 3090 GPU (24 GB memory), Intel Xeon Gold 6248R CPU, and 128 GB RAM. All baseline models are re-implemented uniformly under the experimental framework of this paper, adopting a consistent data preprocessing pipeline, the same input sample length (1024), the same batch size (64), and the same maximum number of training epochs (100 epochs). The network structures and key hyperparameters of each model strictly follow the recommended settings in their original literature. Each set of experiments is run independently 5 times, and the mean and standard deviation are reported to evaluate the statistical stability of the results.

### Comparison with baselines

**Fig. 2:**
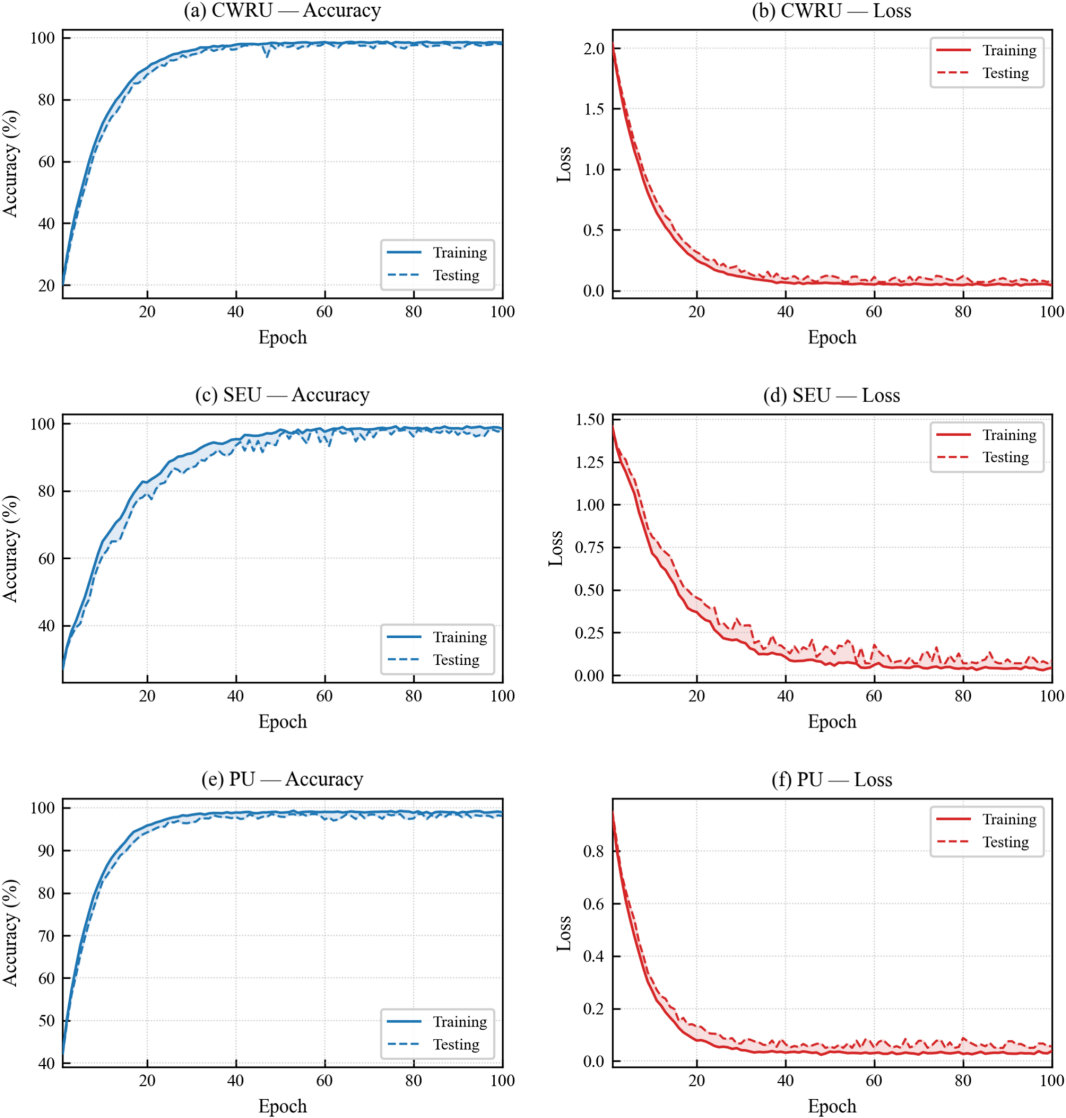
Training accuracy and loss curves on three datasets.

Before comparing the diagnostic accuracy of different models, the training stability of MDM-CA Net is first evaluated. Figure 2 shows the training and test curves of the proposed method on the CWRU, SEU, and PU datasets. It can be observed that the model exhibits good convergence characteristics across different operating conditions and datasets. On the CWRU dataset (Fig. 2a,b), both the training and test accuracy curves rise rapidly within the first few epochs and then level off. On the more complex SEU dataset with varying rotational speeds (Fig. 2c,d), despite some oscillations during adjustment, the model still achieves effective convergence within approximately 40 epochs. On the PU dataset (Fig. 2e,f), the model also demonstrates stable convergence behavior. Overall, the training and test curves remain closely aligned without noticeable divergence, indicating that the adaptive cross-attention mechanism and regularization strategies such as Dropout effectively suppress overfitting, ensuring the model’s generalization capability on unseen data.

1. Experimental results on the CWRU dataset

As shown in Table 4, on the CWRU dataset, which has a relatively high signal-to-noise ratio and relatively single operating conditions, all deep learning models achieve high diagnostic accuracy. MDM-CA Net achieves the overall best performance with an accuracy of 97.68% and an F1-score of 97.33%. The advantage of the proposed method is particularly significant in recall (97.85%), which is 1.83 percentage points higher than that of CNN-Transformer (96.02%). However, in terms of precision, CNN-Transformer (97.05%) slightly outperforms MDM-CA Net (96.82%). This reveals the different diagnostic tendencies of the two methods: the global self-attention of CNN-Transformer tends toward conservative discrimination to reduce false alarms, whereas the cross-attention mechanism of MDM-CA Net tends to actively detect potential faults, achieving a lower missed diagnosis rate at the cost of a small number of false alarms. For safety-critical industrial monitoring scenarios, this high-recall-oriented diagnostic strategy holds greater practical value.

**Table 4:** Performance comparison of models on the CWRU dataset (%).

Model	Accuracy	Precision	Recall	F1-score
1DCNN	93.25±0.58	93.42±0.62	93.08±0.60	93.25±0.59
BiGRU	93.87±0.53	93.62±0.57	94.15±0.55	93.88±0.54
GCN	95.12±0.47	95.38±0.51	94.82±0.49	95.10±0.48
GAT	94.78±0.46	94.45±0.50	95.15±0.48	94.80±0.47
DRSN	95.62±0.43	95.18±0.46	96.05±0.44	95.61±0.44
DSCNN-BiGRU	95.35±0.41	95.85±0.44	94.88±0.42	95.36±0.42
CNN-Transformer	96.53±0.37	97.05±0.40	96.02±0.39	96.53±0.38
MDM-CA Net	97.68±0.29	96.82±0.33	97.85±0.31	97.33±0.30

(2) Experimental results on the SEU dataset

As shown in Table 5, in the SEU cross-domain test with varying rotational speeds, operating condition drift and strong background noise significantly impair the generalization capability of the models. Two unsupervised domain adaptation baselines, DANN and CORAL, are additionally introduced in this experiment to evaluate the performance difference between specialized domain adaptation methods and the proposed dynamic manifold reconstruction strategy.

Among the baseline models, although DANN (93.68%) and CORAL (93.35%) outperform the basic models without domain adaptation, neither exceeds the accuracy of the graph neural network baseline (GCN: 94.36%), indicating that in cross-domain bearing fault diagnosis scenarios, topology-based feature modeling may be more effective than explicit distribution alignment. Consistent with the observations on the CWRU dataset, the same cross-pattern persists among the baselines—GCN outperforms GAT, and DRSN outperforms DSCNN-BiGRU—confirming that these cross-metric patterns are inherent characteristics of architectural inductive biases. Among all baselines, CNN-Transformer achieves the best overall performance (accuracy 96.15%).

MDM-CA Net achieves an accuracy of 97.62% and an F1-score of 97.66% on the SEU dataset, attaining the global optimum across all four metrics. This represents an improvement of approximately 1.5 percentage points over CNN-Transformer, with standard deviations reduced to ± 0.35–0.39. Notably, unlike on the CWRU dataset where CNN-Transformer held a slight advantage in precision, MDM-CA Net achieves comprehensive superiority in the SEU cross-domain scenario. This indicates that the multi-view dynamic manifold reconstruction mechanism, by integrating complementary topological information from both the time and frequency domains, can more effectively reconstruct the feature manifold structure under operating condition drift, thereby simultaneously improving detection sensitivity and discrimination accuracy.

**Table 5:** Performance comparison of models on the SEU dataset (%).

Model	Accuracy	Precision	Recall	F1-score
1DCNN	92.45±0.71	92.68±0.76	92.12±0.74	92.40±0.73
BiGRU	93.12±0.65	92.85±0.69	93.42±0.67	93.13±0.66
DANN	93.68±0.62	93.95±0.66	93.28±0.64	93.61±0.63
CORAL	93.35±0.64	93.08±0.68	93.65±0.65	93.36±0.65
GCN	94.36±0.56	94.58±0.60	94.08±0.58	94.33±0.57
GAT	94.15±0.54	93.82±0.58	94.52±0.56	94.17±0.55
DRSN	95.23±0.49	94.82±0.53	95.65±0.50	95.23±0.49
DSCNN-BiGRU	94.88±0.47	95.38±0.50	94.42±0.48	94.90±0.49
CNN-Transformer	96.15±0.42	96.52±0.45	95.78±0.43	96.15±0.44
MDM-CA Net	97.62±0.35	97.45±0.39	97.88±0.37	97.66±0.36

(3) Experimental results on the PU dataset

As shown in Table 6, since the PU dataset contains only three health states, the classification difficulty is relatively low, and the overall accuracy of all models is higher than that on CWRU and SEU. DRSN achieves the highest recall among all models on the PU dataset (98.17%), surpassing that of MDM-CA Net (97.83%). The PU dataset contains real damages generated from accelerated lifetime tests, whose fault impulse morphologies are more complex and diffuse than those of artificial damages. The soft-thresholding mechanism of DRSN behaves more “aggressively” when processing such signals, preserving weak impulses as much as possible by lowering the threshold, thereby achieving an extremely high detection rate—but at the cost of a significant decrease in precision (95.50% vs. 98.33% of MDM-CA Net). MDM-CA Net still achieves the best overall performance with an accuracy of 98.00% and an F1-score of 98.08%, demonstrating its more balanced diagnostic strategy between precision and recall.

Considering the results across the three datasets, MDM-CA Net exhibits differentiated performance characteristics: on CWRU, its weakness lies in precision (lower than CNN-Transformer); on PU, its weakness lies in recall (lower than DRSN); and on the cross-domain SEU dataset, it achieves comprehensive superiority. This cross-dataset differentiated performance indicates that MDM-CA Net does not pursue extreme optimization in a single direction but adaptively seeks the optimal balance point in the precision-recall space according to the data characteristics—a property of great significance for practical deployment scenarios that require adaptation to multiple operating conditions.

**Table 6:** Performance comparison of models on the PU dataset (%).

Model	Accuracy	Precision	Recall	F1-score
1DCNN	94.17±0.55	94.33±0.59	93.83±0.57	94.08±0.56
BiGRU	94.83±0.51	94.50±0.55	95.17±0.53	94.83±0.52
GCN	95.67±0.45	95.92±0.48	95.33±0.47	95.62±0.46
GAT	95.33±0.44	95.08±0.48	95.67±0.45	95.37±0.45
DRSN	96.33±0.41	95.50±0.45	98.17±0.38	96.82±0.40
DSCNN-BiGRU	96.00±0.40	96.50±0.43	95.50±0.41	96.00±0.41
CNN-Transformer	97.17±0.35	97.50±0.38	96.83±0.37	97.16±0.36
MDM-CA Net	98.00±0.28	98.33±0.30	97.83±0.32	98.08±0.29

(4) Confusion matrix analysis

To deeply analyze the classification details, this section selects the SEU dataset for confusion matrix comparison. This dataset adopts a cross-domain setting (training on Condition 1, testing on Condition 2), where the combination of domain shift and strong background noise makes it the most challenging scenario among the three datasets in terms of generalization. Moreover, SEU contains five health states with relatively high feature similarity among fault types, facilitating the revelation of differences in fine-grained fault identification through confusion matrices. The best-performing baseline among the nine models, CNN-Transformer, is selected for comparison.

**Fig. 3:**
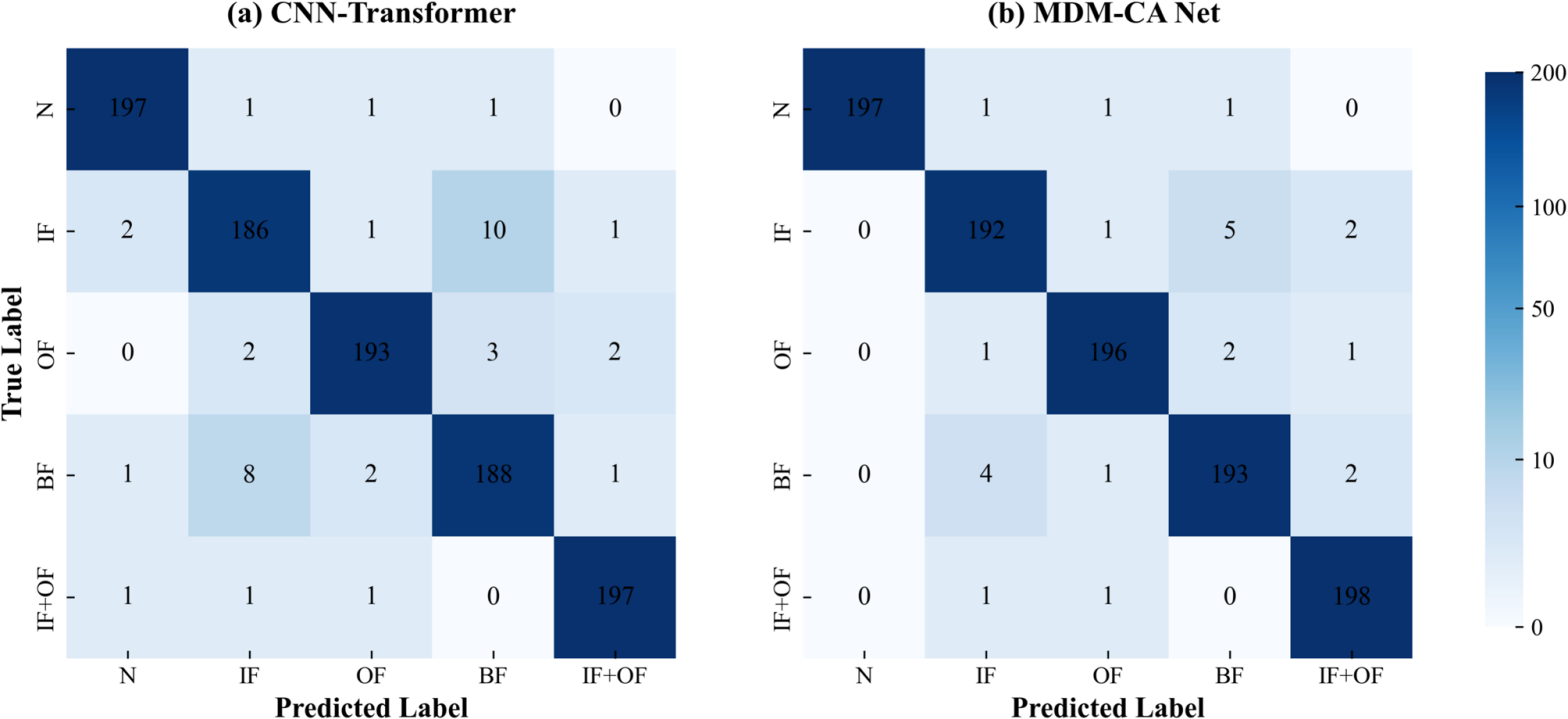
Confusion matrix analysis on the SEU dataset.

As shown in Fig. 3a, the main misclassifications of CNN-Transformer are concentrated between inner race fault (IF) and ball fault (BF), exhibiting significant bidirectional confusion: 10 IF samples are misclassified as BF, and 8 BF samples are misclassified as IF. Additionally, a total of 4 fault samples (2 IF, 1 BF, 1 IF+OF) are missed and misclassified as normal. Such “fault $\rightarrow$ normal” missed detections pose a direct risk to industrial safety.

As shown in Fig. 3b, MDM-CA Net significantly alleviates the above two types of misclassifications. On one hand, the number of mutually confused samples between IF and BF is reduced from 18 (10 + 8) to 9 (5 + 4), a reduction of 50%, indicating that the multi-view dynamic manifold reconstruction effectively captures the subtle differences between these two fault types in the frequency-domain topology. On the other hand, the first column of the confusion matrix (predicted as normal) is completely cleared, meaning that no fault sample is misdiagnosed as normal. This implies that in this test, MDM-CA Net correctly identifies all fault samples as “abnormal,” achieving zero missed detections. For safety-critical industrial monitoring tasks, this zero-missed-detection characteristic holds significant practical value.

(5) Computational cost analysis

To evaluate the engineering deployment feasibility of each model, Table 7 systematically compares the computational costs of the models from four dimensions: number of parameters, floating-point operations (FLOPs), training time, and single-sample inference latency. It should be noted that DANN and CORAL, as unsupervised domain adaptation methods, involve their domain discriminator (DANN) and covariance alignment loss (CORAL) only during the training phase. Their inference-stage forward paths are identical to that of their backbone network, 1DCNN. Therefore, the inference-stage parameter counts, FLOPs, and inference latency of these two models are the same as those of 1DCNN and are not listed separately.

1DCNN holds a lightweight advantage with 0.31M parameters and an inference latency of 0.83 ms. MDM-CA Net has 2.13M parameters and an inference latency of 4.56 ms, making it the most computationally expensive model among all those compared. This overhead primarily stems from the computation of the $N \times N$ distance matrix in the dual-channel dynamic K-NN graph construction and the matrix multiplication operations in the cross-attention module. Nevertheless, an inference latency of 4.56 ms remains acceptable for most industrial online monitoring scenarios. Future research can further reduce the computational cost through model compression techniques such as pruning, knowledge distillation, or sparse attention to better suit edge deployment scenarios.

In summary, at the cost of a certain computational overhead, MDM-CA Net achieves significant improvements in diagnostic accuracy and robustness, making it suitable for safety-critical equipment monitoring tasks where high reliability is paramount.

**Table 7:** Computational cost comparison of all models on the CWRU dataset.

Model	Params (M)	FLOPs (M)	Training time (s/epoch)	Inference latency (ms/sample)
1DCNN	0.31	15.2	12.5	0.83
BiGRU	0.52	28.6	18.4	1.13
GCN	0.89	52.3	25.7	2.10
GAT	1.12	68.5	31.5	2.74
DRSN	1.56	85.1	29.1	2.32
DSCNN-BiGRU	1.45	76.4	28.9	2.47
CNN-Transformer	1.78	112.7	36.1	3.26
MDM-CA Net	2.13	156.3	42.2	4.56

### Noise robustness experiments

To validate the robustness of the proposed method under low signal-to-noise ratio (SNR) conditions, additive white Gaussian noise (AWGN) of varying intensities is added to the original vibration signal *x*(*t*). The noisy signal $\hat{x}(t)$ is defined as:

16 $$\begin{aligned} \hat{x}(t)=x(t)+n(t) \end{aligned}$$

where *n*(*t*) is a Gaussian white noise sequence, and the noise intensity is controlled by the signal-to-noise ratio:

17 $$\begin{aligned} \textrm{SNR}_{\textrm{dB}}=10 \log _{10}\left( \frac{P_{\text{ signal } }}{P_{\text{ noise } }}\right) \end{aligned}$$

Experiments are conducted under six different SNR levels: SNR = − 4, − 2, 0, 2, 4, 6 dB. MDM-CA Net and all baseline models are systematically tested on the CWRU, SEU, and PU datasets. It should be noted that DANN and CORAL only participate in the noise experiments on the SEU dataset and are not included in the noise tests on CWRU and PU.

1. Experimental results with Gaussian white noise

**Fig. 4:**
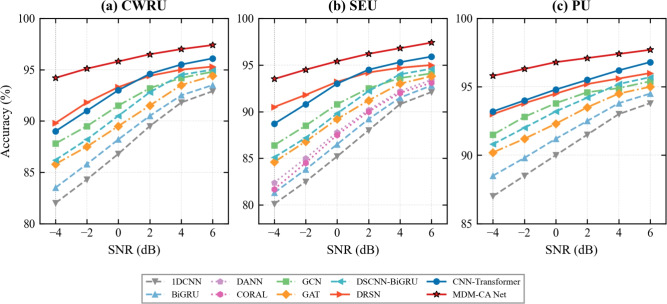
Accuracy under AWGN at varying SNR levels on three datasets.

Figure 4 shows the accuracy curves of the models as a function of SNR on the three datasets. The following key findings can be summarized:

Robustness advantage of MDM-CA Net: MDM-CA Net maintains leading accuracy across all SNR intervals and all datasets, with the most gradual performance degradation curve. Specifically, on the CWRU dataset (Fig. 4a), the accuracy of the model decreases smoothly from 96.5% at 6 dB to 94.2% at − 4 dB, with a total drop of only 2.3 percentage points. Under the same conditions, the second-best baseline, DRSN, drops from 94.5% to 89.8%, a decline of 4.7 percentage points—approximately twice that of MDM-CA Net. On the cross-domain SEU dataset (Fig. 4b), MDM-CA Net decreases from 96.2% at 6 dB to 93.5% at − 4 dB, a drop of 2.7 percentage points, while DRSN drops from 93.8% to 90.5%, a decline of 3.3 percentage points. On the PU dataset (Fig. 4c), MDM-CA Net decreases from 97.2% at 6 dB to 95.8% at − 4 dB, a drop of only 1.4 percentage points, whereas the second-best baseline, CNN-Transformer, drops from 96.0% to 93.2%, a decline of 2.8 percentage points.

These results indicate that the performance advantage of MDM-CA Net expands significantly as noise intensity increases: at 6 dB, the advantage over the second-best baseline is approximately 1.2–1.5 percentage points; at − 4 dB, this advantage widens to 2.6–4.4 percentage points. This characteristic—“the stronger the noise, the greater the relative advantage”—demonstrates that the multi-view dynamic manifold reconstruction mechanism can effectively leverage complementary topological information from the time and frequency domains to filter noise and prevent the collapse of the feature space.

Ranking changes among baseline models: In the high SNR range (4–6 dB), the relative ranking of the baselines is largely consistent with the noise-free results shown in Tables 4, 5 and 6, with CNN-Transformer maintaining the best performance among the baselines due to its powerful feature extraction capability. However, as noise intensity increases, dataset-dependent differentiation in the baseline rankings emerges. On the CWRU and SEU datasets, DRSN surpasses CNN-Transformer at approximately 0–2 dB, becoming the best-performing baseline under noisy conditions. This is because the self-attention weights of CNN-Transformer are easily dominated by noise components at low SNR, leading to degradation of its global modeling capability. In contrast, the soft-thresholding module of DRSN adaptively suppresses noise amplitudes, exhibiting greater resilience under extreme noise. On the PU dataset, which contains only three categories and relatively good signal quality, the performance gap between the two is small across all SNR ranges, with CNN-Transformer maintaining a slight lead over DRSN throughout. Furthermore, the simple concatenation fusion strategy of DSCNN-BiGRU fails more rapidly as noise intensifies; its accuracy is overtaken by GCN at approximately 2–4 dB, indicating that shallow fusion is less effective than a single-stream graph convolutional model under strong noise environments.

(2) Experimental results with non-Gaussian noise

Noise interference in real industrial environments extends far beyond the Gaussian white noise form. To more comprehensively evaluate the robustness of the models, this section further introduces two types of industrially representative non-Gaussian noise on the SEU dataset (cross-domain setting) for testing:

Impulsive noise: Simulates transient large-amplitude interference in scenarios such as equipment collisions and gear meshing impacts. It is generated using an $\alpha$-stable distribution model (characteristic exponent $\alpha$ = 1.5), with amplitude calibrated according to the equivalent SNR.

Colored noise: Simulates low-frequency environmental vibrations and structural resonances commonly found in mechanical systems. It is generated using a pink noise model whose power spectral density is proportional to 1/f.

Table 8 shows the accuracy comparison of the models under three noise types at the extreme noise condition of -4 dB.

**Table 8:** Accuracy (%) under different noise types on the SEU dataset at − 4 dB SNR.

Model	AWGN	Impulse noise	Colored noise
1DCNN	80.1±0.93	75.7±1.14	83.4±0.87
BiGRU	81.3±0.86	78.1±1.07	84.2±0.79
DANN	82.4±0.81	77.6±1.19	84.7±0.74
CORAL	81.7±0.84	79.1±0.96	83.6±0.82
GCN	86.4±0.67	81.3±0.94	88.3±0.61
GAT	84.6±0.72	79.8±1.03	87.1±0.66
DRSN	90.5±0.53	87.8±0.71	91.6±0.49
DSCNN-BiGRU	85.1±0.69	81.7±0.88	87.9±0.64
CNN-Transformer	88.7±0.58	85.3±0.76	91.9±0.51
MDM-CA Net	93.5±0.42	91.4±0.53	94.8±0.38

From Table 8, the following key observations can be drawn:

Differential impact of noise types on model performance: All models perform significantly worse under impulsive noise than under AWGN and colored noise, while achieving the best performance under colored noise. Impulsive noise appears as large-amplitude transient disturbances, directly disrupting the local structure of the time-domain waveform and causing a global impact on feature extraction. Colored noise (pink noise) has its energy concentrated in the low-frequency band, whereas the characteristic frequencies excited by bearing faults are typically located in the mid-to-high frequency range, resulting in natural separability in the frequency domain. Therefore, most models generally perform better under colored noise than under AWGN.

Further analysis of the relative performance degradation from AWGN to impulsive noise reveals differences in the resistance of different architectures to extreme transient interference. Models with built-in denoising or gating mechanisms exhibit stronger resilience: MDM-CA Net shows the smallest relative degradation (2.2%), followed by DRSN (3.0%), both significantly lower than models such as GCN (5.9%), GAT (5.7%), and DANN (5.8%). Notably, the graph topology-based models GCN and GAT suffer severe performance loss under impulsive noise, with relative degradation as high as 5.9% and 5.7%, respectively. This is because impulsive noise introduces large-magnitude outliers in the feature space, directly distorting the adjacency structure of the K-NN graph and generating numerous spurious connections between normal samples and noise points. As a result, the graph convolution aggregates contaminated neighborhood information, leading to severe degradation of topological features. In contrast, the dynamic K-NN graph construction mechanism of MDM-CA Net can adaptively identify and reject outlier nodes on a per-sample basis. Moreover, the complementary information from multiple views provides redundancy validation, effectively preventing the collapse of static graph topology under impulsive noise.

Noise sensitivity analysis of domain adaptation models: DANN exhibits a particularly pronounced performance degradation under impulsive noise (an absolute drop of 4.8 percentage points and a relative drop of 5.8%), ranking second only to GCN among all models. The adversarial training process makes it more difficult for the feature extractor and domain discriminator of DANN to maintain the adversarial equilibrium under extreme disturbances, thereby significantly weakening the stability of the learned feature representations. In contrast, CORAL performs implicit domain adaptation simply by minimizing the difference in covariance matrices, without involving an adversarial game during training, and thus demonstrates stronger stability under impulsive noise (relative degradation of 3.2%). This difference indicates that under extreme noise conditions, the training mechanism of domain adaptation methods has a non-negligible impact on robustness: although adversarial training-based methods can learn stronger domain-invariant features under normal conditions, the fragility of their training equilibrium may become a weakness when noise surges.

Ranking changes among baseline models: Under AWGN conditions, DRSN achieves the highest accuracy among the baselines (90.5%) due to its soft-thresholding denoising capability, outperforming CNN-Transformer (88.7%). This ranking is maintained under impulsive noise (DRSN 87.8% > CNN-Transformer 85.3%), as the thresholding mechanism of DRSN can suppress large-amplitude impulses, preserving a certain denoising advantage. However, under colored noise, the ranking reverses: CNN-Transformer (91.9%) surpasses DRSN (91.6%). This is because colored noise has a distinct frequency structure, and the global self-attention of CNN-Transformer can precisely distinguish low-frequency interference from mid-to-high frequency fault features. In contrast, the soft-thresholding operation of DRSN performs uniform compression based on amplitude, lacking selectivity in the frequency domain and thus failing to fully exploit the natural separability between colored noise and fault features in the frequency distribution. Furthermore, as noted earlier, GCN and DSCNN-BiGRU also exhibit a ranking reversal under impulsive noise. These cross-noise-type ranking shifts indicate that the inductive biases of different architectures exhibit differentiated advantages and disadvantages under specific noise conditions, with no single baseline model maintaining a consistently dominant position across all noise types. MDM-CA Net maintains the highest accuracy and the lowest standard deviation under all three noise types, demonstrating the comprehensive robustness of the multi-view dynamic manifold reconstruction and cross-attention fusion against diverse noise interferences.

### Ablation studies

To further validate the effectiveness of each core module in MDM-CA Net and its contribution to the overall performance, a series of ablation experiments are conducted on the SEU dataset (cross-domain setting). Five model variants (M1–M5) are constructed by progressively removing or replacing key components to evaluate their impact. The definitions of the variants are as follows:

M1 (baseline): A basic dual-stream network. It uses a static K-NN graph to construct the spatial stream, employs BiGRU to extract the temporal stream, and performs fusion via simple feature concatenation.

M2 (w/o dynamic): Removes the dynamic graph construction mechanism. A fixed global K-NN graph is pre-constructed before training and remains unchanged during testing. This variant aims to validate the adaptability of dynamic manifold reconstruction to non-stationary signals.

M3 (w/o multi-view): Removes the multi-view strategy. Only time-domain signals are used to construct a single-view K-NN graph, ignoring frequency-domain topological information. This variant aims to validate the importance of multi-view complementary information in filtering spurious connections.

M4 (w/o cross-Attn): Removes the cross-attention fusion. It retains multi-view dynamic graph construction and temporal enhancement modules but replaces the adaptive cross-attention module with simple feature concatenation. This variant aims to validate the necessity of deep spatiotemporal interaction.

M5 (proposed): The complete MDM-CA Net model as proposed in this paper.

Each variant is run independently five times, and the mean and standard deviation are reported. The experimental results are shown in Table 9.

**Table 9:** Ablation study results for different variants on the SEU dataset (%).

Variant	Dynamic	Multi-view	Cross-Attn	Accuracy	Precision	Recall	F1-score
M1	$\times$	$\times$	$\times$	93.48±0.63	93.62±0.68	93.25±0.66	93.43±0.65
M2	$\times$	$\surd$	$\surd$	95.15±0.52	95.32±0.56	94.95±0.54	95.13±0.53
M3	$\surd$	$\times$	$\surd$	95.85±0.47	95.42±0.52	96.28±0.49	95.85±0.48
M4	$\surd$	$\surd$	$\times$	96.25±0.43	96.68±0.46	95.82±0.45	96.25±0.44
M5	$\surd$	$\surd$	$\surd$	97.62±0.35	97.45±0.39	97.88±0.37	97.66±0.36

Based on the experimental results, the following three conclusions can be drawn:

The central role of dynamic manifold reconstruction (M1 vs. M2 & M5): Compared to the baseline model M1 (93.48±0.63%), M2—which incorporates multi-view and cross-attention mechanisms but retains a static graph—achieves an improved accuracy of 95.15±0.52% (+1.67%). However, this remains significantly lower than that of the full model M5 (97.62±0.35%). The only difference between M2 and M5 lies in the adoption of a dynamic graph construction mechanism. The performance gap of 2.47 percentage points strongly indicates that under varying operating conditions or strong noise, the manifold structure of the signal evolves dynamically over time. A static graph fails to capture such topological changes; consequently, even when equipped with powerful components such as multi-view and cross-attention, its performance remains fundamentally constrained. In contrast, the dynamic reconstruction mechanism adaptively updates the adjacency relationships on a per-sample basis, thereby enabling a more accurate characterization of the fault evolution trajectory.

Complementarity of multi-view information and active retrieval capability of cross-attention (M3 vs. M4): In M3 and M4, the multi-view strategy and the cross-attention module are removed, respectively. Their overall accuracies are comparable (95.85±0.47% vs. 96.25±0.43%), yet they exhibit significant complementary crossovers in fine-grained metrics. M4, leveraging multi-view graph construction to provide higher-quality spatial features, achieves a precision of 96.68±0.46%, substantially higher than that of M3 (95.42±0.52%). This indicates that the topologically complementary information from the time and frequency domains can effectively eliminate spurious connections in a single domain and reduce false alarms. However, in terms of recall, M3 (96.28±0.49%) surpasses M4 (95.82±0.45%), revealing the unique value of the cross-attention module: through its active retrieval mechanism of “temporal querying–spatial retrieval,” the model is able to proactively detect potential fault samples even when the spatial features are suboptimal (i.e., single-view).

Synergistic enhancement effect (M5 vs. other variants): The complete model M5 achieves globally optimal performance across all four metrics, with its accuracy (97.62±0.35%) significantly surpassing that of any variant lacking a single component. The independent contributions of the three core modules are as follows: dynamic graph construction contributes + 2.47% (M2$\rightarrow$M5), multi-view strategy + 1.77% (M3$\rightarrow$M5), and cross-attention +1.37% (M4$\rightarrow$M5). Notably, the synergistic gain of all three modules (M1$\rightarrow$M5: + 4.14%) exceeds the simple additive sum of the contributions of any two components. This superlinear synergy indicates a positive mutual reinforcement among the modules: when multi-view reconstruction is absent (M3), cross-attention is easily misled by noisy spurious connections in a single view; when cross-attention is absent (M4), high-quality spatial features fail to couple nonlinearly with temporal evolution information. Examining the trend in standard deviations, from M1 to M5, the standard deviation gradually decreases from ±0.63 to ±0.35, demonstrating that the introduction of each core module not only improves diagnostic accuracy but also enhances the statistical stability of the results. Only when all three components work synergistically can the MDM-CA Net achieve effective decoupling of complex compound fault features under cross-operating-condition scenarios with extremely low signal-to-noise ratios.

### Feature visualization

To intuitively evaluate the discriminative ability of features extracted by different models, the t-SNE method is employed to project high-dimensional features into a two-dimensional space for visualization. Since the CWRU dataset has a relatively high signal-to-noise ratio and involves only a single operating condition, most comparative models achieve fairly good feature separation, making it difficult to capture subtle differences in feature extraction capability among various methods. Therefore, this subsection focuses on presenting the feature distribution comparison on the SEU gearbox dataset (under variable-speed operating conditions). This dataset not only contains strong background noise but also exhibits significant operating condition drift, imposing higher demands on model robustness.

It should be noted that the two domain adaptation baselines, DANN and CORAL, achieve accuracies (93.68% and 93.35%, respectively) close to that of BiGRU (93.12%). Preliminary analysis indicates that their feature distribution patterns resemble that of BiGRU, all showing notable inter-class overlap. Due to space limitations in the manuscript, Figure 6 does not present the t-SNE results of these two models individually; instead, it focuses on representative comparisons across different architectural types.

**Fig. 5:**
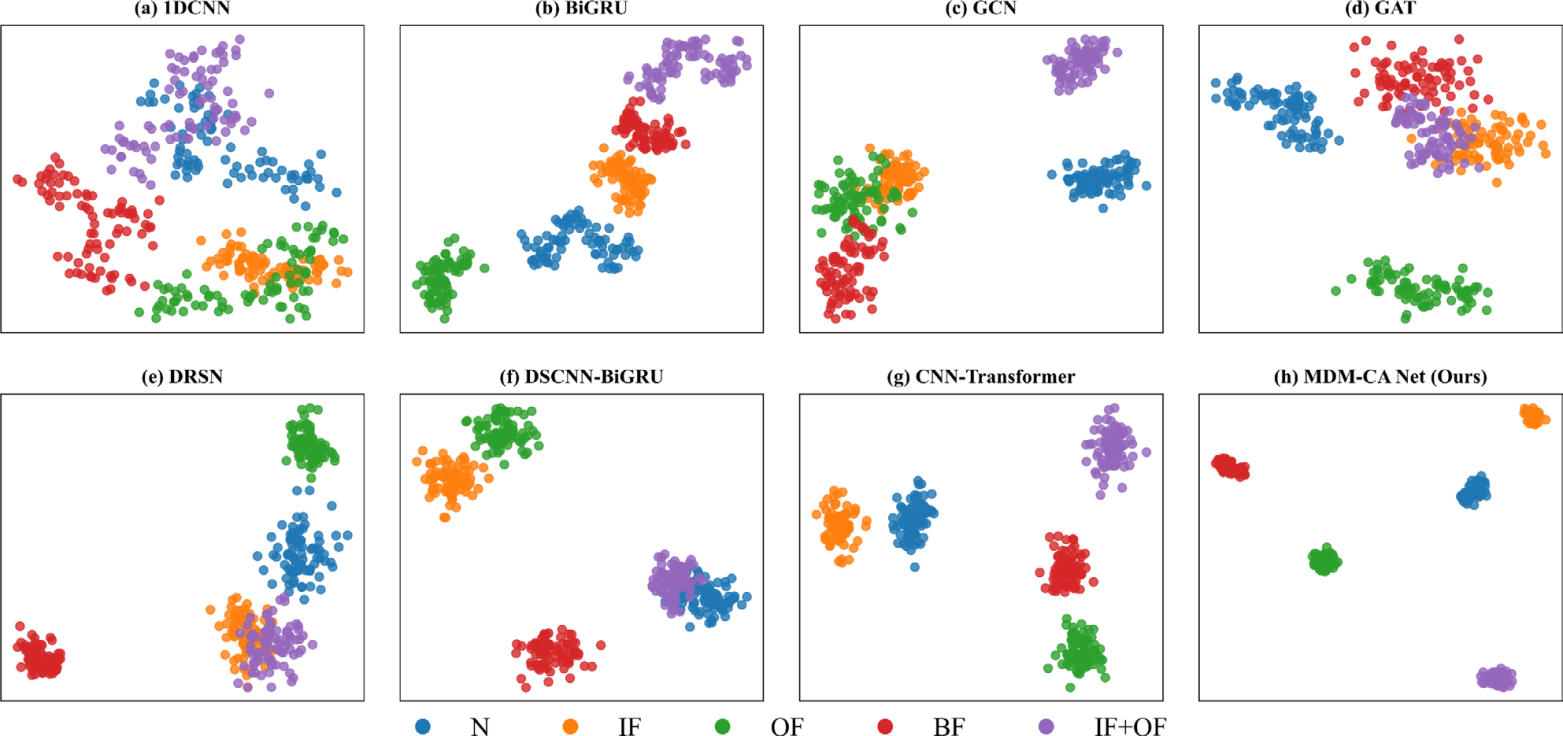
t-SNE feature visualization analysis on the SEU dataset.

As shown in Fig. 5a,b, under variable operating condition interference, the feature spaces of the baseline models (1DCNN, BiGRU) exhibit pronounced disorder. The feature clusters of different fault categories are severely overlapped and entangled, indicating that the models fail to effectively decouple complex fault patterns. With improved network architectures (Fig. 5c,d), graph neural networks (GCN, GAT) achieve somewhat better clustering performance, yet noticeable adhesion remains at the inter-class boundaries, and some outliers deviate from the main clusters. This suggests that the topological quality of single-view graph construction is limited under noisy environments. DRSN (Fig. 5e) suppresses part of the noise interference via its soft-thresholding mechanism, leading to improved feature separability; however, the intra-class distributions remain insufficiently compact. DSCNN-BiGRU and CNN-Transformer (Fig. 5f,g) demonstrate favorable separation capability, with each fault category forming relatively distinct and well-separated clusters. Nevertheless, their intra-cluster distributions are still not compact enough, with a small number of sample points lying outside the main clusters, indicating limited capability in suppressing intra-class variability.

In contrast, the proposed MDM-CA Net (Fig. 5h) demonstrates optimal feature extraction performance. The feature clusters of different fault categories exhibit excellent intra-class compactness and inter-class separability, with virtually no cross-class outliers. This indicates that the multi-view dynamic manifold reconstruction and cross-attention mechanism effectively mine the intrinsic topological structure of the data, significantly enhancing feature robustness. The source of this advantage can be further understood through the ablation analysis: the multi-view mechanism filters out spurious connections via time–frequency complementarity, thereby improving the purity of the feature clusters (corresponding to high precision); the cross-attention mechanism actively retrieves key nodes within the spatial topology, enhancing the assignment of marginal samples to the correct fault categories (corresponding to high recall). Working in synergy, these two mechanisms enable the feature space of MDM-CA Net to achieve both compact intra-class aggregation and clear inter-class separation.

## Conclusion

This paper proposes a bearing fault diagnosis method, termed MDM-CA Net, which integrates multi-view dynamic manifold reconstruction and a cross-attention mechanism. The method achieves robust diagnosis under complex operating conditions through three synergistic core modules: a dynamic K-NN graph construction mechanism that adaptively updates the manifold topology on a per-sample basis to track the feature evolution trajectory of non-stationary signals; a multi-view strategy that simultaneously exploits complementary topological information from the time and frequency domains to filter out spurious connections in a single domain; and a cross-attention fusion module that enables deep spatiotemporal interaction via an active retrieval strategy of “temporal querying–spatial retrieval.” Systematic experiments on three public bearing datasets—CWRU, SEU, and PU—validate the effectiveness of the proposed method. Under both same-condition (CWRU, PU) and cross-condition (SEU) scenarios, MDM-CA Net achieves the best overall diagnostic performance. Notably, under cross-condition scenarios, it significantly outperforms classic domain adaptation methods such as DANN and CORAL, indicating that dynamic manifold reconstruction is more effective than explicit distribution alignment in mitigating domain shift. Ablation studies reveal the synergistic enhancement effect among the modules: the multi-view mechanism drives high precision, while the cross-attention mechanism drives high recall; only when working in synergy can the two achieve optimal balance. Noise robustness experiments show that MDM-CA Net maintains stable performance advantages under three types of interference—Gaussian white noise, impulsive noise, and colored noise—with the advantage becoming more pronounced as noise intensity increases, thereby validating the structural immunity of the dynamic manifold reconstruction mechanism to noise.

The main limitation of the proposed method lies in its computational cost: the dual-channel dynamic K-NN graph construction and the cross-attention module result in an inference latency of 4.56 ms. Although this is acceptable for most industrial scenarios, it does not yet meet the millisecond-level real-time requirements for edge deployment. Future work will focus on the following directions: (1) reducing computational overhead through model compression techniques such as pruning, knowledge distillation, or sparse attention, while introducing an incremental manifold update strategy to replace per-sample full reconstruction, thereby adapting to streaming data for edge deployment; (2) introducing a transfer validation protocol that accounts for cross-device, cross-condition, and sensor bias variations, incorporating domain adaptation and transfer learning strategies to evaluate the generalization stability of the proposed method when there are larger distribution discrepancies between the training and test domains; (3) extending the robustness study to a broader range of industrial interference types, systematically assessing the impact of sensor bias, compound interference, and real-world mechanical disturbances on the stability of manifold reconstruction; (4) generalizing the proposed method to fault diagnosis tasks of other rotating machinery, such as gearboxes and electric motors, to further validate its versatility.

## Supplementary Information

Supplementary Information.

## Data Availability

The CWRU and SEU datasets used in this study are publicly available from their respective sources. The CWRU dataset is available from https://engineering.case.edu/bearingdatacenter/apparatus-and-procedures. The SEU dataset is available from https://github.com/cathysiyu/Mechanical-datasets/tree/master/gearbox.
