# Supplementary material for: Multi-view dynamic manifold reconstruction with adaptive cross-attention fusion enables noise-robust bearing fault diagnosis
Source: Sci Rep. 2026 Apr 29;16:20007. doi: 10.1038/s41598-026-50621-z (PMC13319751; doi:10.1038/s41598-026-50621-z)
Supplement: Supplementary file 1 — Supplementary Information. [file 41598_2026_50621_MOESM1_ESM.pdf]

Supplementary Information

The effectiveness of the proposed dynamic graph construction strategy is predicated on the assumption that the KNN algorithm can correctly identify true neighbors. Under low signal-to-noise ratio (SNR) conditions, noise distorts the Euclidean distances between nodes, thereby affecting neighbor identification. The following analysis examines the stability of the KNN adjacency matrix defined by Equation (5) under additive noise from three perspectives.

(1) Statistical stability of distance ranking. Let the noise-free node features be $\mathbf{x}_i \in \mathbb{R}^{d_{in}}$. After adding additive white Gaussian noise (AWGN), we have $\tilde{\mathbf{x}}_i = \mathbf{x}_i + \varepsilon_i$, where $\varepsilon_i \sim \mathscr{N}(0, \sigma_n^2 \mathbf{I})$. The squared distance of a noisy node pair (i, j) can be expanded as:

$$\tilde{d}_{ij}^2 = d_{ij}^2 + 2(\mathbf{x}_i - \mathbf{x}_j)^T(\varepsilon_i - \varepsilon_j) + \|\varepsilon_i - \varepsilon_j\|^2 \tag{1}$$

where $d_{ij} = \|\mathbf{x}_i - \mathbf{x}_j\|$ is the true distance. The expectation of the third term $\|\varepsilon_i - \varepsilon_j\|^2$ is $2d_{in}\sigma_n^2$, which is independent of the node pair (i, j) and thus introduces an approximately uniform global shift across all distances. Owing to the phenomenon of concentration of measure in high-dimensional spaces, when d_{in} is sufficiently large, the fluctuation of this term across different node pairs is extremely small. Since the KNN algorithm relies on the relative ranking of distances rather than their absolute values, a global shift does not alter the ranking. Consequently, the relative ordering of neighbors remains statistically stable.

(2) Adaptive error tolerance of the Gaussian kernel. For a small number of false neighbors that enter the K-nearest neighbor set due to noise perturbation, their distances are typically significantly larger than those of true neighbors. The Gaussian kernel $\exp(-\tilde{d}_{ij}^2/\sigma^2)$ in Equation (5) causes the weights of these spurious edges to decay exponentially. After the normalized neighborhood aggregation in Equation (6), the informational contribution of spurious edges is suppressed to a negligible level, thereby endowing the adjacency matrix with inherent tolerance to occasional graph construction errors.

(3) Cross-validation via multi-view complementarity. Additive noise ε acts uniformly on each sampling point in the time domain. After FFT transformation, Parseval's theorem ensures $\|\hat{\varepsilon}\| = \|\varepsilon\|$, meaning the total noise energy is conserved. However, the distribution of signal energy differs fundamentally between the two domains: bearing fault characteristic frequencies (e.g., f_{BPFI}, f_{BPFO}, and their harmonics) are sparsely concentrated in the frequency domain, whereas AWGN remains a uniformly flat spectrum in the frequency domain. Consequently, the frequency-domain view achieves a local signal-to-noise ratio in the fault-characteristic frequency bands that is significantly higher than the global SNR, making KNN graph construction more accurate within these subspaces. In contrast, the time-domain view is more sensitive to transient impact pulses induced by faults, maintaining high graph construction quality within the local time windows where such impacts occur. Since the perturbations of distance rankings caused by noise in the two domains are statistically uncorrelated, the neighbor relationships corrupted in the time domain have little systematic overlap with those corrupted in the frequency domain. The learnable parameter λ in Equation (7) automatically adjusts the contribution weights of the two views, allowing correct graph information to compensate for each other during the fusion stage, thereby forming a cross-validation effect. This analysis is consistent with the significant performance degradation observed in the ablation variant M3 (which removes the multi-view strategy).

In summary, the noise robustness of the dynamic KNN graph construction stems from the synergistic interplay of three mechanisms: ranking stability, Gaussian kernel attenuation, and multi-view cross-validation.
